# Orthogonal Demodulation Pound–Drever–Hall Technique for Ultra-Low Detection Limit Pressure Sensing

**DOI:** 10.3390/s19143223

**Published:** 2019-07-22

**Authors:** Jinliang Hu, Sheng Liu, Xiang Wu, Liying Liu, Lei Xu

**Affiliations:** 1 Key Lab for Micro and Nanophotonic Structures (Ministry of Education), Department of Optical Science and Engineering, School of Information Science and Engineering, Fudan University, Shanghai 200433, China; 2 Department of Physics, Fudan University, Shanghai 200433, China

**Keywords:** whispering-gallery mode (WGM) microcavity, micro-bubble resonator, Pound–Drever–Hall technique, pressure sensing

## Abstract

We report on a novel optical microcavity sensing scheme by using the orthogonal demodulation Pound–Drever–Hall (PDH) technique. We found that larger sensitivity in a broad range of cavity quality factor (Q) could be obtained. Taking microbubble resonator (MBR) pressure sensing as an example, a lower detection limit than the conventional wavelength shift detection method was achieved. When the MBR cavity Q is about 10^5^–10^6^, the technique can decrease the detection limit by one or two orders of magnitude. The pressure-frequency sensitivity is 11.6 GHz/bar at wavelength of 850 nm, and its detection limit can approach 0.0515 mbar. This technique can also be applied to other kinds of microcavity sensors to improve sensing performance.

## 1. Introduction

The Pound–Drever–Hall (PDH) technique [1,2] is a high-sensitivity wavelength sensing technique that locks the wavelength of a laser to the resonant mode of a high-quality factor (Q) reference cavity. A single frequency laser generates sidebands after passing a phase modulator and launches into a reference cavity; when the laser wavelength is detuned from the resonance, the de-modulated detection technique generates a sharp bipolar voltage-wavelength discriminating signal. The slope of the discriminating signal, which determines the wavelength sensitivity, depends on the Q of mode and the phase modulation frequency. For a sharp resonance with Q > 10^6^ and phase modulation frequency larger than 50 MHz, the wavelength sensitivity of PDH can be significantly larger than directly monitoring the resonant wavelength change. The PDH technique utilizes the lock-in technique that can avoid the influence of light intensity perturbation to generate a higher signal-to-noise ratio (SNR) discriminating signal. Due to the high sensitivity and SNR [2,3,4] of PDH technique, studies on vibration detection [5], strain sensing [5,6,7,8,9], microcavity gyroscope [4,10], particle detection [11,12], and pressure tuning and sensing [13] have been published. Up to now, PDH sensing demodulation is processed by the lock-in technique, and the modulated output is directly mixed with the local oscillation, which also drives the phase modulator. However, when the phase modulation frequency exceeds 10 MHz, there will be an unknown but constant phase difference between the detection signal and the local oscillation drive, which will have a significant influence on achieving sharp slope discriminating signal.

Over the past decade, whispering gallery mode (WGM) optical cavities [14] have attracted much attention because of specific features such as high intensity, small mode volumes (V), and high Q. Optical microcavities have become a perfect platform to study the microcavity sensor [15,16,17,18,19], optomechanics [20], cavity quantum electrodynamics (CQED) [14], nonlinear optics [21], microcavity lasers [22], optical signal processing [23,24], and single molecule detection [11,12,15,16,17]. The microbubble resonator (MBR) is a new, important type of high-Q microcavity that can be easily used in optofluidic performance [18,19,22], refractometry, aqueous sensing [25], and aerostatic pressure sensing [26,27,28,29] because of its hollow structure. Pressure sensing is an important application for MBRs. Decreasing the wall thickness is an efficient way to decrease the detection limit (DL). Henze et al. [28] used a silica MBR with a wall thickness of 2.9 µm and a diameter of 384 µm to achieve a sensitivity of 22 GHz/bar at wavelength of 780 nm. Madugani et al. [13] used the PDH technique to tune the laser in an MBR and validate pressure sensing, its sensitivity was 5.8 GHz/bar, and the DL was 2 mbar. Later on, Yang et al. [29] prepared an ultrathin-walled thickness (~ 500 nm) MBR and obtained a sensitivity of 30 GHz/bar at a wavelength of 780 nm and its DL was 0.17 mbar. 

In this work, we report that by using orthogonal PDH demodulation, higher wavelength sensitivity can be achieved through a wide range of cavity Q at high phase modulation frequency. No random phase reduction is required. On the other hand, it is not necessary to prepare ultrathin-walled MBRs to achieve high sensitivity. The pressure-frequency sensitivity is 11.6 GHz/bar at a wavelength of 850 nm, and its DL can approach 0.0515 mbar.

## 2. Experimental Setup and Principle

The WGM resonators in the experiments are MBRs, fabricated by heating pressurized silica capillaries with a fiber fusion splicer. A tiny change of MBR size or refractive index in the hollow core can cause a shift of resonant frequency, which can be used for sensing. 

**Figure 1:**
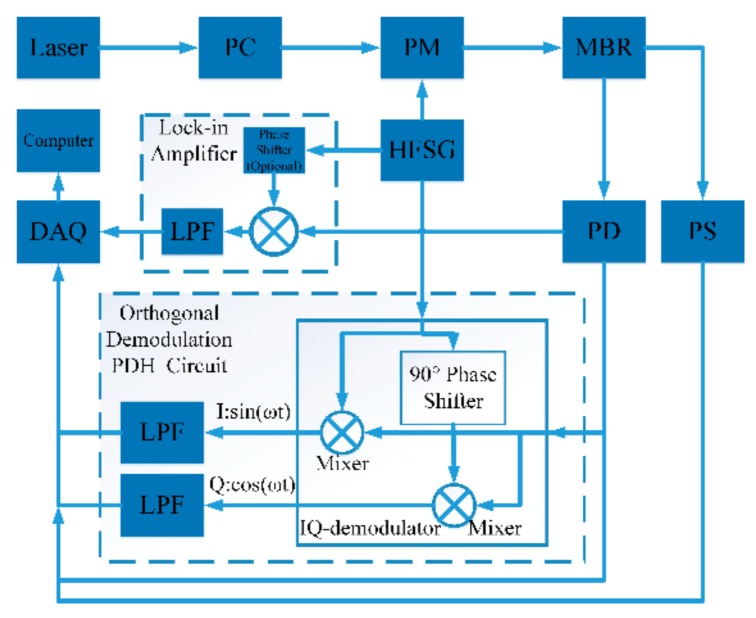
Schematic setup of lock-in and orthogonal demodulation Pound–Drever–Hall (PDH) technique with 850 nm tunable laser (New Focus TLB 6716). PC, polarization controller; PM, phase electro-optical modulator (iXBlue NIR-MPX800); MBR, micro-bubble resonator; PD, photoelectric detector (Thorlabs PDA10A2); LPF, low-pass filter; DAQ, NI data acquisition card (NI PCIe 6351); HFSG, high frequency function signal generator; PS, pressure sensor.

Figure 1 shows the schematic setup of the lock-in and orthogonal demodulation PDH technique. It can also be used to monitor mode resonant frequency shift. To excite modes of MBR, an 850 nm tunable laser source passing through the polarization controller and phase electro-optical modulator (EOM) is coupled into and out of the MBR via a tapered fiber with a diameter of about 2 μm. The experimental setup is placed in a constant temperature environment to avoid thermal tuning. The transmission light is detected by a photon detector (PD) connected to the data acquisition card (DAQ) and IQ demodulation circuits. A pressure sensor monitors the MBR internal pressure to calibrate the MBR pressure sensor. The DAQ records the signals from the PD, IQ demodulation circuits, and pressure sensor. The DAQ generates a triangular wave signal to sweep the laser frequency. Because the laser frequency sweeps linearly with triangular wave signal, it changes linearly with time in half of the period. Conventionally, PDH uses a lock-in amplifier to demodulate the detection signal. Before demodulation, the detection signal can be expressed as [1,2]

(1) $$V(\omega )\;=I_{0}(\omega )\cos(\omega_{m}t+\varphi )+Q_{0}(\omega )\sin(\omega_{m}t+\varphi ).$$

where *ω* is the laser angular frequency; *ω*_m_ is the angular frequency of the signal input in the EOM (*ω*_m_ = 2π*f*_m_, *f*_m_ = 50 MHz); *φ* is the phase difference caused by reactance between the local oscillation signal and the detection signal. *I*_0_(*ω*) is the in-phase term; *Q*_0_(*ω*) is the quadrature term. The *I*_0_(*ω*) and *Q*_0_(*ω*) can be expressed as [2]

(2) I0(ω) =2E2J0(β)J1(β)Re[R(ω)R*(ω+ωm)−R*(ω)R(ω−ωm)],

(3) Q0(ω) =2E2J0(β)J1(β)Im[R(ω)R*(ω+ωm)−R*(ω)R(ω−ωm)].

where *R*(*ω*) is the complex transmitted spectrum; *E*^2^ represents the laser power; *J*_0_(*β*) and *J*_1_(*β*) are the 0-order and 1-order Bessel functions, and *β* is the modulation degree. A single mode in MBR can be expressed as

(4) $$R(\omega )=1-\frac{2\omega_{0}/q_{e}}{\omega_{0}/q_{0}+\omega_{0}/q_{e}+2i(\omega -\omega_{0})}.$$

where *ω*_0_ is the mode resonant angular frequency; *q*_0_ is the intracavity Q; and *q*_e_ is the external cavity Q. Generally, the lock-in amplifier demodulates detection signals by mixing *V*(*ω*) with local oscillation after a phase shifter. *I*(*ω*) = ½*I*_0_(*ω*) or *Q*(*ω*) = ½*Q*_0_(*ω*) can be obtained at *φ* = 0 and *φ* = π/2 respectively. Here, we use the orthogonal demodulation that IQ demodulator generates *I*(*ω*) and *Q*(*ω*) simultaneously by using the IQ-demodulator, which does not require phase shifter (to set *φ* = 0 and *φ* = π/2). Using orthogonal oscillation (sin(*ω*_m_*t*) and cos(*ω*_m_*t*)) to demodulate *V*(*ω*), we have

(5) I(ω) =12A0(ω)cos[φ(ω)+φ],

(6) Q(ω) =12A0(ω)sin[φ(ω)+φ],

in which A0(ω)=[I02(ω)+Q02(ω)]12 and $\varphi (\omega )=\arctan\left[Q_{0}(\omega \right)/I_{0}(\omega )]$, obviously,

(7) $$A(\omega )=\left\{\begin{array}{l} \sqrt{I^{2}(\omega )+Q^{2}(\omega )}\;\;\;(I(\omega )\geq 0)\\ -\sqrt{I^{2}(\omega )+Q^{2}(\omega )}\;\;\;(I(\omega )<0) \end{array}\right.$$

does not relate to *φ*(*ω*) and *φ*. 

**Figure 2:**
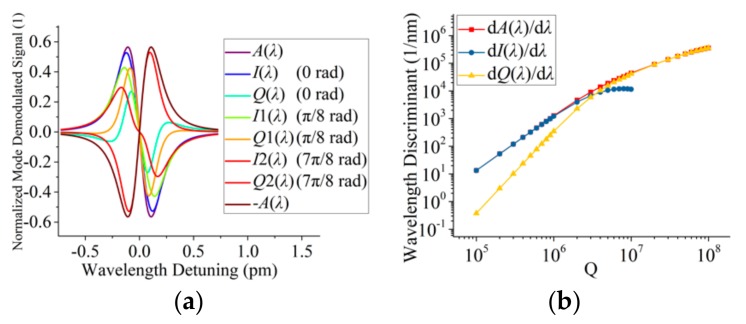
(**a**) Normalized mode demodulated signal when Q is 3 × 10^6^ at a wavelength of 850 nm; (**b**) wavelength discriminant (WD) at different Q near resonant wavelength at a wavelength of 850 nm.

Figure 2a illustrates a simulated demodulation result of a normalized mode when *Q* = 3 × 10^6^ (*Q* = (*q*_0_^−1^ + *q*_e_^−1^)^−1^) at a wavelength of 850 nm; *φ* is set at 0, π/8, and 7π/8, respectively. The y-axis represents the normalized demodulated signal from normalized mode as a function of wavelength at a resonant wavelength of 850 nm. Although the signal is a normalized result, the signal maximum cannot reach unit 1 because of low Q; it can reach unit 1 at high Q, which is a property of discriminating wavelength signal [2]. The discriminating wavelength signal near the resonant wavelength is linear with the wavelength. The slope of the discriminating wavelength signal near the resonant wavelength is called the wavelength discriminant (WD, *WD*_I_ = d*I*(*λ*)/d(*λ*), *WD*_Q_ = d*Q*(*λ*)/d(*λ*), *WD*_A_ = d*A*(*λ*)/d(*λ*)) [2]. WD is the discriminating wavelength sensitivity. *φ* = 0 represents the ideal condition. On the other hand, *φ* = π/8 and 7π/8 represent non-ideal conditions. Obviously, *A*(*λ*) has the largest WD in the three cases. We chose the phase (π/8) to demonstrate that phase can influence WD significantly and the phase (7π/8) to demonstrate the phase can influence the lineshape direction of I-term and Q-term signals. Figure 2b plots simulation results of *WD*_I_, *WD*_Q_, and *WD*_A_ as a function of Q at a wavelength of 850 nm; *φ* is set at 0. *WD*_I_, *WD*_Q_, and *WD*_A_ increases as Q increases. *WD*_I_ is larger than the *WD*_Q_ when Q is below 2 × 10^6^. When Q is above 2 × 10^6^, *WD*_Q_ is larger than *WD*_I_. Saturation occurs when the modulation frequency is approaching the bandwidth of the resonant mode, therefore sidebands can be hardly modulated [2]. As shown in Figure 2b, *A*(*λ*) has the largest WD in a very large range of cavity Q, which is a perfect property to improve sensor sensitivity.

**Figure 3:**
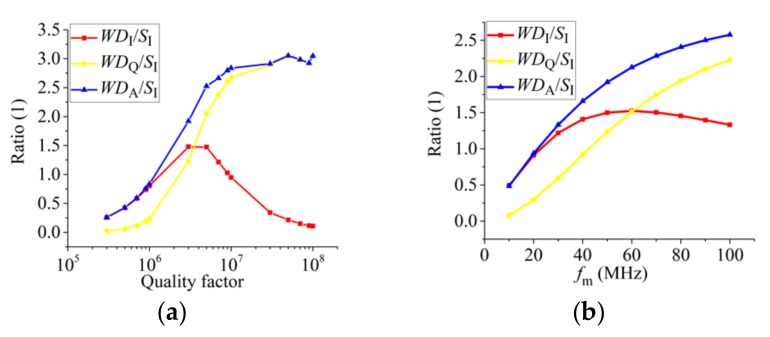
Normalized mode ratio of (**a**) WD and sensitivity of light intensity at different Q when *f*_m_ = 50 MHz; (**b**) WD and maximal sensitivity of light intensity at different modulation frequency when *Q* = 3 × 10^6^.

We also simulated the ratio of WD and maximal light transmission intensity sensitivity (MLTIS, *S*_I_ = (d*I*/d*λ*)_max_) at different Q values and modulation frequencies at a wavelength of 850 nm, illustrated in Figure 3. MLTIS is the max slope of the resonance lineshape. The three curves in Figure 3a,b have the same variation trends. As modulation frequency and Q increase, we find that *WD*_A_/*S*_I_ is always larger than the others. *WD*_I_/*S*_I_ will increase at first and then decrease. *WD*_Q_/*S*_I_ is smaller than *WD*_A_/*S*_I_ but increases continuously. The main reason of *WD*_I_/*S*_I_ and *WD*_Q_/*S*_I_ following different trends is the different responses of I-term and Q-term at low- and high-quality factor region. When the bandwidth of mode is much larger than EOM modulation frequency, almost only the I-term signal (cos term) exists; the Q-term signal (sin term) exists weakly; *WD*_I_ is larger than *WD*_Q_. On the other hand, when EOM modulation frequency is much larger than the bandwidth of the mode, almost only the Q-term signal (sin term) exists; the I-term signal (cos term) exists weakly; *WD*_Q_ is larger than *WD*_I_ [2]. In the simulation of Figure 3b, the Q of the mode is 3 × 10^6^, and its FWHM is about 117 MHz. The modulation frequency is not much larger than the FWHM, thus *WD*_A_/*S*_I_ does not saturate. *WD*_A_/*S*_I_ can be 2–3 when the Q of mode is 2 × 10^6^–10^7^. *WD*_A_/*S*_I_ can be 2–2.5 when the modulation frequency is 50–100 MHz.

We fit the MBR transmitted spectrum with a Lorentzian lineshape function to get the resonance parameters, such as resonant frequency (*f*_0_) and mode FWHM (Δ*f*). We use the following equation [30] to estimate the Q:

(8) $$Q=f_{0}/\Delta f.$$

The use of pressure (*p*) sensing by monitoring the MBR resonant wavelength shift is well known [26]. We use the DAQ to control the tunable laser sweeping the MBR and record the transmitted spectrum and pressure sensor signals continuously. We change the MBR internal pressure stepwise. We use the Lorentzian lineshape function to fit the resonance to obtain the resonant wavelength; we plot the resonant wavelength and pressure data in one figure. The slope of the curve is the pressure sensitivity (*S*_w_). When pressure changes, the MBRs resonant wavelength shifts for two reasons, the MBR refractive index (RI) and size change caused by stress. As a result, the pressure sensitivity (*S*_w_) by monitoring wavelength shift can be written as [26,28,31,32]

(9) $$S_{w}=\frac{d\lambda }{dp}=\lambda \left(\frac{3C}{n_{0}}+\frac{4G+3K}{12GK}\right)\chi ,$$

where *χ* = *b*^3^/(*a*^3^−*b*^3^). *a* and *b* are the outer and inner radius of a MBR shell, and *n*_0_ is the RI of the MBR. The elastic-optic, shear and bulk moduli constants of fused silica are *C* = 4 × 10^-12^ m^2^/N, *G* = 31 × 10^9^ Pa, and *K* = 41 × 10^9^ Pa, respectively. *c* is light velocity in vacuum, and *λ* is the laser wavelength in vacuum. Equation (9) reveals that decreasing the MBR wall thickness can effectively improve the sensitivity. Wavelength shift DL can be defined as

(10) $$DL_{1}=3\sigma /S_{w}.$$

where *σ* is the standard deviation (SD) of resonant wavelength, and *S*_w_ is resonant wavelength shift sensitivity. Pressure sensing by PDH uses a laser with fixed wavelength and purges air into the MBR to change internal pressure. We use the DAQ to record the signals from the IQ demodulator and pressure sensor signal continuously. The relative shift between laser wavelength and MBR resonant wavelength does generate the sharp bipolar voltage-wavelength discriminating signal. In the experiment, when the laser wavelength was fixed and the resonance shifted as the pressure changed, the discriminating signal represented MBR resonance shift near the resonant wavelength. We plotted the discriminating signal and pressure data in one figure. The slope of the curve is the pressure sensitivity (*S*_PDH_). The pressure sensitivity (*S*_PDH_) by PDH near the resonant frequency can be defined as

(11) $$S_{\mathrm{PDH}}=\frac{dA(\lambda )}{dp}=\frac{dA(\lambda )}{d\lambda }\frac{d\lambda }{dp}=S_{p}S_{w}.$$

The sensitivity can be divided into two parts: Resonant wavelength shift sensitivity (*S*_w_ = d*λ*/d*p*), and the voltage-wavelength sensitivity (*S*_p_ = d*A*(*λ*)/d*λ*) caused by the PDH system. The PDH method DL can be defined as

(12) $$DL_{2}=3\sigma_{1}/S_{\mathrm{PDH}}.$$

where *σ*_1_ is the SD of the measured voltage signal. The SDs represent the noise of two experimental setups; however, the measurands of the two setups are different; SDs cannot be compared directly. Therefore, we can use Equation (13) as a transition to obtain the equivalent SD and compare the noise of two setups.

(13) $$\sigma =k\sigma_{1}$$

where *k* (24 pm/V) is the conversion factor.

## 3. Results and Discussion

**Figure 4:**
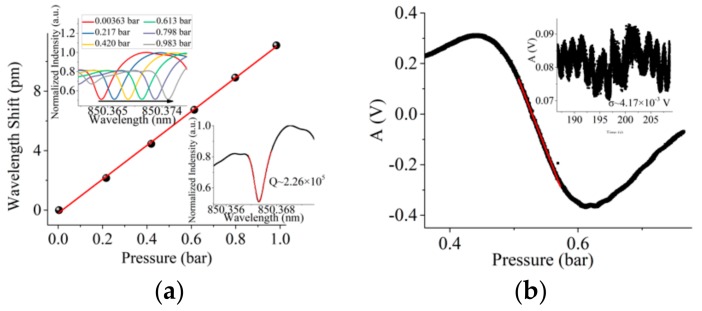
(**a**) Wavelength shift sensing results; insert illustrates *Q* = 2.26 × 10^5^ and the process of wavelength shift; (**b**) orthogonal demodulation PDH sensing results; insert illustrates system noise.

**Figure 5:**
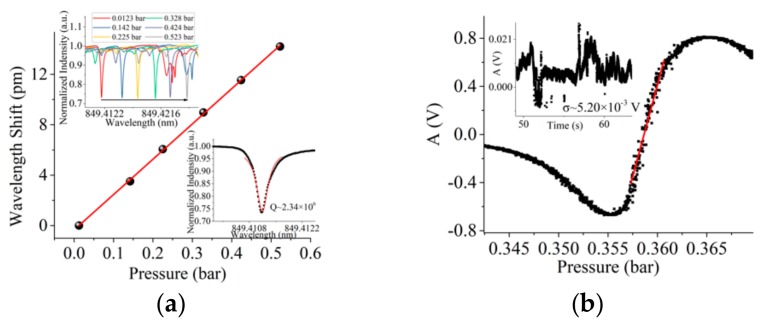
(**a**) Wavelength shift sensing results. insert illustrates *Q* = 2.34 × 10^6^ and the process of wavelength shift; (**b**) orthogonal demodulation PDH sensing results. The insert illustrates the system noise.

Fast sweep (triangular wave signal frequency of 10 Hz) is used to avoid the thermal effect in the experiment. Figure 4a illustrates the pressure sensing result when *Q* = 2.26 × 10^5^. The MBR has a diameter of 332 µm and a wall thickness of 9 µm. The laser power is about 3.13 mW (driving current is 80 mA). Considering the loss of EOM (3.5dB), the power input into the MBR is about 1.40 mW. Pressure sensitivity (*S*_w_) is 0.0114 nm/bar (4.73 GHz/bar). SD of the wavelength shift (*σ*) is 6.25 × 10^−5^ nm. According to Equation (10), DL is 1.64 × 10^−2^ bar. In the insert of Figure 4a, the mode is asymmetric. Usually optical resonant modes of MBR are very complex [33,34]. Resonances overlap each other, which leads to asymmetric mode. Because of the low Q mode and low power laser, we do not think that thermal effect is a big issue. Figure 4b illustrates the sensing result by the orthogonal demodulation PDH method. Pressure sensitivity (*S*_PDH_) by orthogonal demodulation is 6.47 V/bar. SD of the voltage signal (*σ*_1_) is 4.17 × 10^−3^ V (equivalent SD is 1.00 × 10^−4^ nm). According to Equation (12), DL is 1.93 × 10^−3^ bar, one order of magnitude lower than that sensed by wavelength shift. Figure 5a illustrates the sensing result when *Q* = 2.34 × 10^6^. The MBR has a diameter of 276 µm and a wall thickness of 4 µm. The laser power is about 1.94 mW (driving current is 60 mA). Considering the loss of EOM (3.5 dB), the power input into the MBR is about 0.863 mW. Pressure sensitivity (*S*_w_) is 0.0280 nm/bar (11.6 GHz/bar). SD of the wavelength shift (*σ*) is 6.01 × 10^−5^ nm. According to Equation (10), DL is 6.44 × 10^−3^ bar. Figure 5b illustrates the sensing result by the orthogonal demodulation. Pressure sensitivity (*S*_PDH_) by orthogonal demodulation PDH method is 303 V/bar. SD of the voltage signal (*σ*_1_) is 5.20 × 10^−3^ V (equivalent SD is 1.25 × 10^−4^ nm). In the insert of Figure 5b, the comparison and root square operation in Equation (7) will lead to non-white noise. According to Equation (12), DL is 5.15 × 10^−5^ bar. Two order of magnitude lower than that sensed by wavelength shift. The DL is better than in a previous study [29] without ultrathin-walled MBR. The noise levels of the two methods are close, but the sensitivity is significantly different. The sensitivity of the PDH method is improved by the voltage-wavelength sensitivity, as is shown in Figure 2b, so the DL will decrease a lot. 

Our results show that for a higher Q mode, the orthogonal PDH technique can lower the DL more because higher Q leads to a sharper discrimination slope. Meanwhile, for resonant mode shift detection, if Q is over 1 × 10^6^, the DL depends mainly on noise and sensitivity, rather than on Q [35]. Further increasing Q to increase the WD and replacing the IQ-demodulation circuits by a vector network analyzer (VNA) to decrease the noise will help to decrease the DL.

## 4. Conclusions

In summary, we built an orthogonal demodulation PDH sensing system, and achieved 5.15 × 10^−5^ bar DL in pressure sensing at a wavelength of 850 nm without ultrathin-walled MBR. The orthogonal demodulation PDH sensing technique can improve the DL by about one or two orders of magnitude when Q is about 10^5^–10^6^. It can be applied to all kinds of microcavity sensors based on the resonant wavelength shift mechanism to improve performance.
